# Pregnancy related breast diseases in a developing African country: Initial Sonographic Evaluation

**DOI:** 10.11604/pamj.2015.20.239.4830

**Published:** 2015-03-13

**Authors:** Adenike Temitayo Adeniji-Sofoluwe, Gbolahan Oladele Obajimi, Millicent Olubunmi Obajimi

**Affiliations:** 1 University of Ibadan, College of Medicine, Department of Radiology & University College Hospital, Ibadan, Nigeria; 2 University of Ibadan, College of Medicine, Department of Obstetrics & Gynaecology and University College Hospital, Ibadan, Nigeria

**Keywords:** Pregnancy, breast, sonographic, African country

## Abstract

Benign diseases are more common than malignant diseases in pregnant and lactating women. Fibroadenomas are the most commonly identified benign breast tumour in pregnant and lactating women. Pregnancy related breast cancer is defined as breast cancer that occurs during pregnancy or within 1 year of delivery. Its incidence is estimated at 1 in 3000 to 1 in 10 000 pregnancies. Several reproductive factors like age at menarche, age at menopause, age at full-term pregnancy, parity, age at any birth and spacing of pregnancies, breast feeding, characteristics of the menstrual cycle, infertility, spontaneous and induced abortions, characteristics of the menstrual cycle and infertility are some of the factors that have been incriminated as risk factors for breast cancer. We sought to describe the predominant breast pattern, sonographic array of pregnancy related breast diseases in women referred to the breast imaging unit in the department of Radiology at the University College Hospital, Ibadan south west Nigeria. Socio-demographic characteristics in these women were also evaluated. Archived images were reviewed and documented and data was analysed with SPSS version 17 and presented with descriptives. In this descriptive study, we retrospectively retrieved the sonomammographic records of 21 women (pregnant or lactating) referred to and imaged in the department of radiology, University college hospital Ibadan, between 2006 and 2013. Diagnostic breast sonograms performed by MO and ATS; Consultant radiologists with 7-10 years’ experience utilized a 7-10 MHz transducer of the General electric GE Logiq P5 machine for the scans. Twenty-one women with ages between 22-42 years (Mean 31.4 ±5.4 SD) pregnant or lactating were referred to the radiology department for sonomammographic evaluation. Majority of the women were in the 3rd decade. Referral was mainly (11) by family Physicians from the general outpatient clinic, 5 were self-referred, 2 from radiotherapy department, 2 from obstetrics and gynaecology department and 1 from the surgical outpatient clinic. Nineteen (89.5%) were lactating and breastfeeding while 2 (10.5%) were pregnant. Nipple discharge (89.5%) was the predominant presenting complaint in the study. They were all married with the majority attaining menarche at age 14.6±2.1 SD years. Most of the women were multi-parous 17(89.5%) and possessed higher level of Education 17 (81.0%). Twenty (96.0%) women had no previous breast disease while only 1 (4.0%) woman had a positive family history of breast cancer. They weighed between 44-102kg (mean 69.84kg±15.33SD). Their mean height was 159.8cm. Waist hip ratio was between 0.69-0.93 (Mean 0.83). The heterogeneous fibroglandular pattern was predominant in 15 (71.4%) women. Final BIRADS assessment of 2 was most frequent (11/21) 52.4% while 19.0% were assigned to BIRADS categories 0 and 1 (4/21). Histological diagnosis of Invasive ductal carcinoma was made in the 3 women with final BIRADS of 5 breast diseases found in most pregnant and lactating women were benign. It is important to note that malignant breast lesions can also occur in this group of women who may assume that the changes noted in their breast are due to lactation.

## Introduction

Breast changes occur during pregnancy and lactation. These changes are physiologic and are due to hormonal influence on the glandular and ductal breast components [1]. Imaging findings in pregnancy and lactation can be demonstrated at breast sonography. These breast findings are different from those in the non-gravid. Generally, there is an increase in the size and density due to increased vascularity to the breast which appears as increased echogenicity [2]. Also tubular hypoechoic structures representing ducts become filled with hypoechoic colostrum and echogenic milk in late pregnancy and during lactation respectively [3, 4]. These sonographic appearances are more marked in the 3rd trimester and early lactation state. But they revert to the pre pregnancy state at cessation of breastfeeding [4]. Due to these changes lactating patients are requested to come for a thorough baseline clinical breast examination at the first obstetric visit [5]. We sought to describe the predominant breast pattern, sonographic array of pregnancy related breast diseases in women referred to the breast imaging unit in the department of Radiology at the University College Hospital, Ibadan south west Nigeria. Socio-demographic characteristics in these women were also evaluated.

## Methods

In this descriptive study, we retrospectively retrieved the radiology request cards and sonomammographic records of 21 pregnant and lactating women referred to and imaged in the department of radiology, University college hospital Ibadan, between 2006 and 2013. Diagnostic breast sonograms were performed by MO and ATS; Consultant radiologists with 7-10 years’ experience. A 7-10 MHz transducer of the General electric GE Logiq P5 machine was utilised for the scans. The final BIRADS assessment using the American College of radiology breast imaging and reporting data system ACR BI-RADS US lexicon [6] assigned was documented. Also archived images were reviewed and documented. Data was analysed with SPSS version 17.

## Results

Twenty-one women with ages between 22-42 years (Mean 31.4 ±5.4 SD) pregnant or lactating were referred to the radiology department for diagnostic sonomammographic evaluation. Majority of the women were in the 3^rd^ decade. Referral was mainly (11) by family physicians from the general outpatient clinic, 5 were self-referred, 2 from radiotherapy department, 2 from obstetrics and gynaecology department and 1 from the surgical outpatient clinic. They were all married with the majority attaining menarche at age 14.6±2.1SD years. None of our patients was postmenopausal. Most of the women were multi-parous 17(89.5%) and possessed higher education 17 (81.0%). They weighed between 44-102kg (mean 69.84kg ±15.33SD). Their mean height was 159.8cm. Waist hip ratio was between 0.69-0.93 (Mean 0.83).

Nineteen (89.5%) were lactating and breastfeeding while 2 (10.5%) were pregnant. Nipple discharge (89.5%) was the predominant presenting complaint in the study. Breast cancer was also diagnosed. Twenty (96.0%) women had no previous breast disease while only 1 (4.0%) woman had a positive family history of breast cancer. The heterogeneous fibroglandular pattern was predominant in 15 (71.4%) women. Table 1 shows that the Final BIRADS assessment of 2 was most frequent (11/21) 52.4% with fibroadenomas, lactating adenomas and mastitis accounting for the majority of diseases in the group. Other benign breast diseases found were abscesses and galactocoeles. Eighteen per cent were assigned to BIRADS categories 0 and 1 (4/21). Histological diagnosis of Invasive ductal carcinoma was made in the 3 women with final BIRADS assessment of 5 (Table 1).

**Table 1:** Final BIRADS assessment categories

Final BIRADS assessment categories	Designation	Frequency	Percent
Inconclusive	0	2	9.0
Normal	1	2	9.0
Benign	2	11	50.0
Probably benign	3	2	9.0
Suspicious	4	1	4.6
Highly Suggestive of Malignancy	5	3	13.6
Known Cancer	6	0	0
**Total**		**21**	

## Discussion

Benign diseases are more common than malignant diseases in pregnant and lactating women [1, 2]. Rusty or milky nipple discharge was the commonest presenting complaint in our study but considered as an occasional complaint in the literature [7]. Another common finding in our patients was duct dilatation with luminal diameters ranging between 4mm-10mm but no intraductal mass was found. Other common findings in the literature are Fibroadenomas and lactating adenomas. These were also found in our study. Fibroadenomas are the most commonly identified benign breast tumour in pregnant and lactating women [7]. Fibroadenomas are frequently present before pregnancy but may not be noticed as they become larger under the influence of hormones associated with pregnancy [7, 8]. Though usually painless, mobile, rubbery and firm, they may also be tender when secondary infarction occurs from rapid over growth of the mass. Fibroadenomas contain both stromal and epithelial components. The connective tissue make up the stroma while the glandular tissue make up the epithelial components. In contrast, lactating adenomas have mainly the epithelial components and little stoma [5, 7].

**Figure 1:**
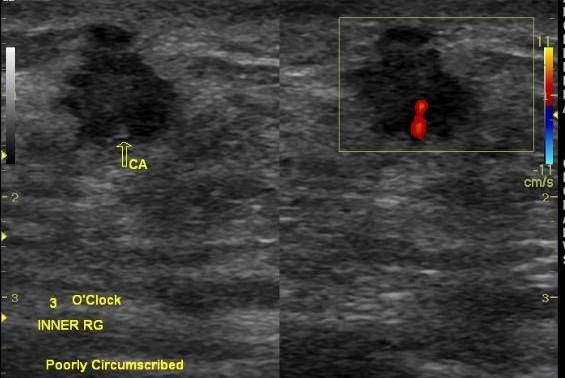
BIRADS 5: (Highly suggestive of Malignancy) Mass on breast Ultrasound

Fibroadenomas are oval, round, wider than tall, well circumscribed masses with 2-3 macrolobulations which is similar to what is found in the non-pregnant or lactating breasts. They may be single/multiple/bilateral. These findings on sonography are indistinguishable from those due to a lactating adenoma but the latter may demonstrate posterior acoustic enhancement due to greater amount of secretions present and may also show increased compressibility [5–8]. Pregnancy related breast cancer is defined as breast cancer that occurs during pregnancy or within 1 year of delivery. Its incidence is estimated at 1 in 3000 to 1 in 10 000 pregnancies [9, 10]. In pregnancy and during lactation, the hormonal changes increase breast size and volume, firmness making clinical and radiologic detection and breast evaluation difficult [10]. We found 3 cases of breast cancer. All had left sided lesions which were hypo echoic, one demonstrated a thick echogenic halo, posterior acoustic shadowing and increased vascularity with Doppler interrogation. One was well circumscribed while 2 were poorly circumscribed (Figure 1). The size of the masses ranged between a width of 2.6-9.1cm and a height of 1.66-3.7cm. There were associated enlarged axillary lymph nodes in the patients with evidence of nodal invasion in only one woman. The women in our study had neoadjuvant and adjuvant chemotherapy. They all had a progression of their disease eventually developing bilateral breast cancer.

The aim of breast cancer treatment in a pregnant or lactating patient is to achieve local control of disease and prevention of systemic metastases. This aim is the same for non-pregnant patients. But some modification is necessary. Neoadjuvant chemotherapy may be suitable because pregnancy related breast cancers usually present at a more locally advanced stage. Also, treatment is such as to minimize harm to the fetus. Therefore, a delay to breast and chest irradiation may be an option until after delivery. The sample size in this study was very small and showed that only few pregnant and lactating women were referred for imaging. Therefore, a larger sample size is required to make more statistically significant observations. The future direction of this study is to utilize ultrasound for the evaluation of the breasts of pregnant women during their visits to the antenatal clinic and the lactating women during their post -natal visit in our institution.

## Conclusion

Abnormal nipple discharge is the most common complaint in pregnancy and lactation. Breast conditions found were benign and related to the pregnant or lactating status. Predominantly duct ectasia was found. It is important to note that malignant breast lesions can also occur in this group of women who may assume that the changes noted in their breast are due to pregnancy and /lactation.
